# Metagenomic analysis of diarrheal stools in Kolkata, India, indicates the possibility of subclinical infection of *Vibrio cholerae* O1

**DOI:** 10.1038/s41598-022-24167-9

**Published:** 2022-11-14

**Authors:** Eizo Takahashi, Daisuke Motooka, Shota Nakamura, Shin-ichi Miyoshi, Goutam Chowdhury, Asish K. Mukhopadhyay, Shanta Dutta, Daichi Morita, Tetsuya Iida, Keinosuke Okamoto

**Affiliations:** 1 Collaborative Research Center of Okayama University for Infectious Diseases in India, NICED-JICA Building, 57 Dr. S.C. Banerjee Road, Beliaghata, Kolkata, 700 010 India; 2 Department of Infection Metagenomics, Research Institute for Microbial Diseases, Osaka University, Suita, Osaka 565-0871 Japan; 3 Center for Infectious Disease Education and Research (CiDER), Osaka University, Suita, Osaka 565-0871 Japan; 4 Graduate School of Medicine, Dentistry and Pharmaceutical Sciences of Okayama University, 1-1-1 Tsushima-Naka, Kita-Ku, Okayama, Okayama 700-8530 Japan; 5 National Institute of Cholera and Enteric Diseases, NICED-JICA Building, 57 Dr. S.C. Banerjee Road, Beliaghata, Kolkata, 700 010 India; 6 Department of Bacterial Infections, Research Institute for Microbial Diseases, Osaka University, Osaka, 565-0871 Japan; 7 Present Address: Department of Health Pharmacy, Yokohama University of Pharmacy, 601 Matano-Cho, Totsuka-Ku, Yokohama, Kanagawa 245-0066 Japan

**Keywords:** Ecology, Microbiology, Gastroenterology

## Abstract

We examined the stools of 23 patients in Kolkata, who were diagnosed as cholera patients because *Vibrio cholerae* O1 was detected from their stools by culturing methods, and further explored by metagenomic sequencing analysis. Subsequently, the presence of the gene encoding A subunit of cholera toxin (*ctxA*) and the cholera toxin (CT) level in these stool samples were examined. *ctxA* was examined by both metagenomic sequencing analysis and polymerase chain reaction. In these examinations, two samples did not show positive in any of these tests. The metagenomic analysis showed that the genes for *Streptococcus pneumoniae* and *Salmonella enterica* were present in the stools of these two patients, respectively. Therefore, these two patients were not considered to have diarrhea due to *V. cholerae* infection. From these results, we predicted that some Kolkata residents harbor a small number of *V. cholerae* in their intestines as a form of subclinical infection with *V. cholerae*. Next, we analyzed the stool samples of 22 diarrhea patients from which *V. cholerae* was not isolated. The results showed that 3 of the patients seemed to have subclinical infection of *V. cholerae* based on the amount of the genes. These results indicated that subclinical infections with *V. cholerae* O1 occur in Kolkata.

## Introduction

In the Kolkata area, many kinds of diarrheagenic microorganisms are present, including bacteria, viruses, and parasites^1,2^. Examination of these microorganisms in the stools of patients is needed to determine the causative microorganism for the diarrhea. In addition, some patients are simultaneously infected with two or more diarrheagenic microorganisms. To cause diarrhea, a minimum number of a diarrheagenic microorganism must be present in the intestinal tract. If the number is too small, the microorganism cannot cause diarrhea. Therefore, the detection of a causative organism of diarrhea in stool is insufficient for determining the etiological microorganism of the diarrhea, and determination of the quantity of the microorganism in the intestinal tract is needed.

Cholera is an extremely serious disease among infectious diarrheal diseases. Cholera is caused by the infection with cholera toxin (CT)-producing *Vibrio cholerae* O1 and *V. cholerae* O139 (toxigenic *V. cholerae*). A diagnosis of cholera is made by isolating toxigenic *V. cholerae* from stool, which is usually achieved by bacteriological examination in the laboratory^1^. However, stools contain many impurities and contaminants that can interfere with the examination. Furthermore, the volume of stool supplied for the examination is small: it is usually 0.5 ml or less. Therefore, it is questionable whether *V. cholerae* is accurately isolated from the stool of every cholera patient.

To improve the efficacy of *V. cholerae* isolation*,* stool samples are often cultured in alkaline peptone water (pH 8.5) to selectively promote the growth of *V. cholerae*^3^*.* In these cultures, the number of toxigenic *V. cholerae* may increase dramatically. Even if the number of toxigenic *V. cholerae* in a sample is very small, it can be isolated easily after the selective culturing, and the patient would be diagnosed as having cholera disease.

It has been reported that the ingestion of a small number of toxigenic *V. cholerae* O1 does not cause cholera symptoms. To cause cholera symptoms, more than a certain number of *V. cholerae* has to settle in the intestinal tract^4,5^.

We considered that it is necessary to analyze quantitatively the mass of *V. cholerae* in the stool of cholera patients to understand the role of the bacteria in causing diarrhea. Here, we performed a quantitative analysis of *V. cholerae* in the stool of 23 cholera patients by metagenomic sequencing. In addition, to examine the characteristics of the *V. cholerae* in the intestinal tracts of these patients, the presence of the cholera toxin A subunit gene (*ctxA*) in stool was examined by polymerase chain reaction (PCR) and by searching the data obtained by metagenomic sequencing analysis. Subsequently, the amount of CT in stool was measured. Furthermore, 22 diarrhea stool samples, from which diarrheagenic microorganisms, including *V. cholerae,* were not detected by routine microbial examination, were analyzed by metagenomic sequencing. These results indicated that *V. cholerae* may spread constantly among people in Kolkata as subclinical infections. We believe that such spreading may be associated with the occurrence of chronic cholera cases in the area.

## Results

### Sample collection and isolation of *V. cholerae* O1 possessing the CT gene

Twenty-three patients (patient numbers 9 to 31) who were diagnosed with cholera were examined in this study. The diagnosis was confirmed by the isolation of *V. cholerae* O1 from the stool of each patient. The age of patients, date of hospital admission, stool sampling date, pathogen isolated and medicines administered to the patients as treatments are described in Supplementary Table S1. Twenty-one of the stool samples were collected on the first day of hospitalization, while the remaining two stool samples were collected on the second day (patient number 29) and fourth day (patient number 10) of hospitalization. All patients had not been given any antibiotics and the samples of diarrheal stool were taken during severe diarrhea.

To confirm the presence of the CT gene (*ctx*) in these 23 isolates, we examined the presence of *ctxA* in these isolates by PCR. The PCR to detect *ctxA* was performed as reported by Keasler and Hall^6^. In this PCR, amplification was performed in 30 cycles. The size of the amplified *ctxA* fragment was 302 bp. The target fragment was amplified from each of the *V. cholerae* O1 isolates. This indicated that all of the *V. cholerae* O1 isolates from the 23 cholera patients possessed *ctxA.*

### CT production from the isolates

**Figure 1:**
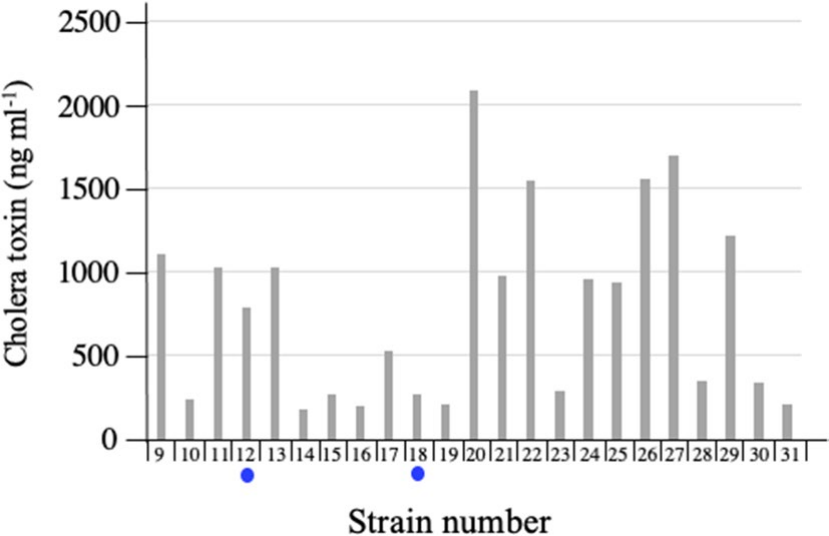
Amount of cholera toxin produced by *V. cholerae* O1 isolated from patients with diarrhea. Twenty-three strains of *V. cholerae* O1 were isolated from 23 patients with diarrhea. These isolates were cultured statically in AKI medium^7^ at 37 °C for 24 h. After removing the cells by centrifugation, the CT in the culture supernatants was measured using a GM1-ganglioside ELISA method^8^. The samples indicated by blue circle are isolates obtained by bacterial culture from two patients (patient 12 and patient 18), who are focused on in this study.

The production of CT from these 23 isolates was examined by detecting secreted CT in the medium. The 23 isolates were cultured statically in AKI medium^7^, and the secreted CT in the culture supernatants was measured using the GM1-ganglioside enzyme-linked immunosorbent assay (ELISA) method^8^. The detection limit of CT by the ELISA method used is 1.0 ng ml^−1^. All the samples examined were found to have CT above this concentration (Fig. 1). This shows that all isolates examined are toxigenic *V. cholerae* O1.

### Analysis of the stool samples of patients diagnosed with cholera disease

#### Metagenomic sequencing analysis

The primary objective of this metagenomic analysis is to show the proportion of *V. cholerae* living in the diarrhea stool. Subsequently, if the number of *V. cholerae* infected in the intestinal tract is small, it is required to clarify the etiological microorganisms that cause diarrhea in that patient. For this analysis, it is necessary to investigate the presence of pathogenic microorganisms other than *V. cholerae* in the stool. To do this, we need to analyze the gene reads obtained by metagenomic analysis with a comprehensive manner. Therefore, we planned to obtain reads with the Burrows-Wheeler Alignment tool (BWA) with default parameters, a matching software with the ability to fulfill these objectives^9^ (http://bio-bwa.sourceforge.net).

However, we were concerned that the genes derived from organisms other than *V. cholerae* in the stool were counted mistakenly as genes derived from *V. cholerae* in the analysis using BWA. We therefore first examined genes in stool from people unrelated to cholera disease to ensure that the analysis method we planned to use in this study would correctly detect genes from *V. cholerae* in stool. For this analysis we used DNA sequences reported by the NIH Human Microbiome Project (https://www.hmpdacc.org/hmp/hmp/hmasm2/). The genes we have analyzed are DNA derived from feces of 20 healthy individuals (10 males and 10 females). The results are shown in Supplementary Table S2.

The number of reads analyzed in this analysis varied from sample to sample. The largest number obtained after quality filtering was 60,975,797. The lowest number was 10,301,809. However, the number of reads detected as originating from *V. cholerae* was very small (12 reads or less) in all samples, and none of them were detected in 7 samples. This very small number shows that the analytical method used is suitable for detecting the genes from *V. cholerae* in these samples.

Therefore, we analyzed DNA and RNA samples from prepared diarrheal stool by the method using BWA. All raw sequencing data obtained were deposited into the DDBJ Sequence Read Archive under the accession code PRJDB10675. This number can be searched not only from DDBJ but also from EMBL and GenBank.

**Figure 2:**
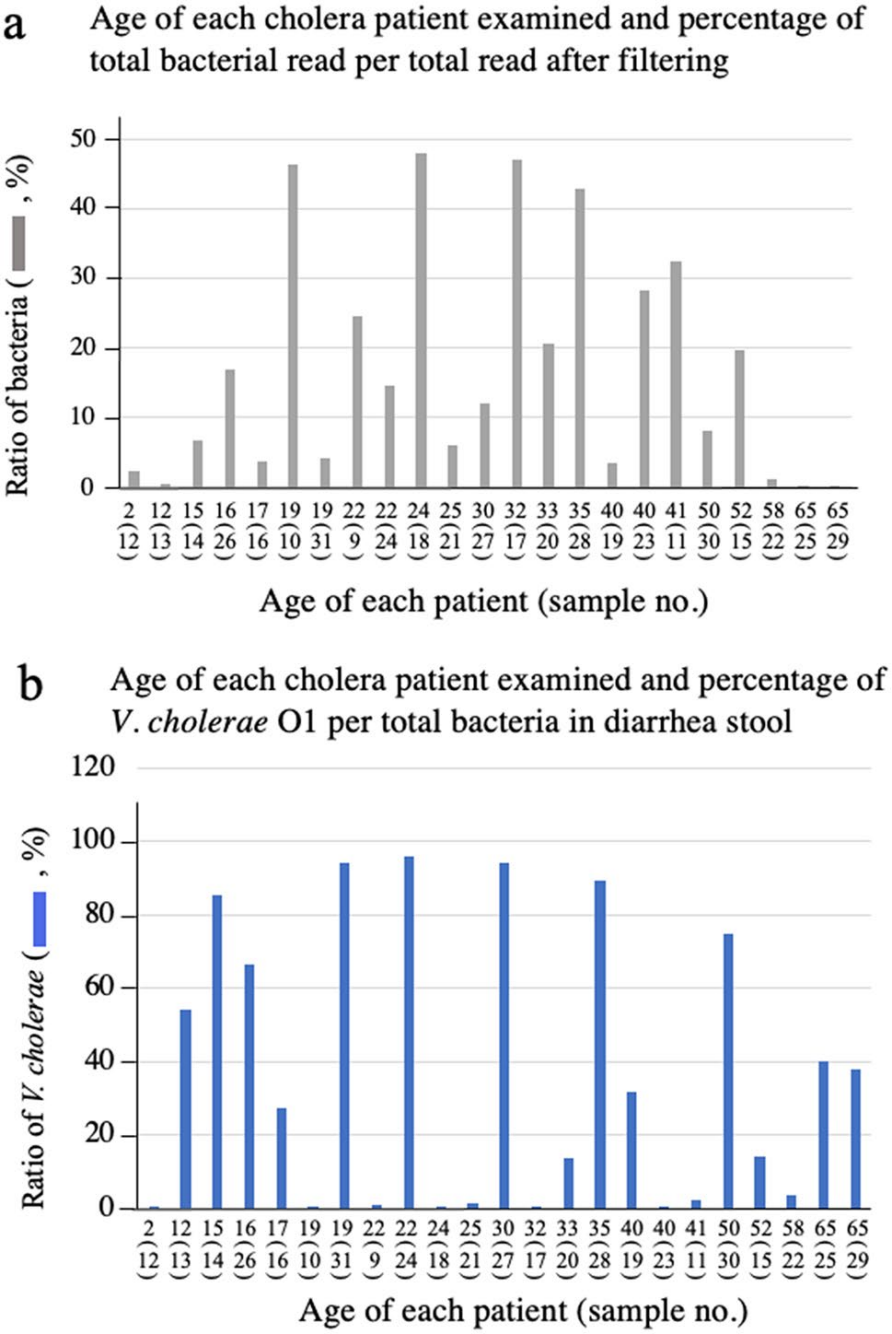
Age of patient and the ratio of the number of read detected by metagenomic sequencing analysis of their stools. The DNA in the stool samples from 23 patients who were diagnosed with cholera disease were extracted using a commercially available kit. Patient ages are listed in Supplementary Table S1. The extracted DNA were investigated by a metagenomic sequencing analysis to clarify the origin of individual DNA. The origin of the DNA sequences was assigned by mapping to a database that included human and microorganism sequences. The obtained numbers of total reads, total bacterial reads, reads originating from *V. cholerae*, reads originating from *ctxA* in each sample are shown in Supplementary Table S3 (the data from DNA sample). The age of each patient and the ratio of the number of reads from all bacteria to the total number of reads after filtering (**a**) and the ratio of the number of reads from *V. cholerae* to the number of reads from all bacteria (**b**) were calculated. The horizontal axis of these figures shows the age of each patient and is the same arrangement in both (**a**) and (**b**). The numbers in parentheses indicate the sample numbers. This sample number is also the patient's number.

Diarrheal stools are mostly composed of liquid, and their properties are very different from those of normal stools. The origin of the nucleic acids in diarrheal stools varies from patient to patient and is not constant. One sample may contain many genes derived from human cells, while another sample may contain many genes derived from microorganisms. To clarify the nature of the reads we obtained, we determined the proportion of reads of bacterial origin to the total number of reads in the samples analyzed, and presented this proportion in order of patient age (Fig. 2a). The ratios were not consistent, indicating that the cells of eukaryotic origin and microorganisms existing in the stool of patients with diarrhea varied from person to person.

This result implied that it was difficult to detect *V. cholerae* in a sample with a small number of read derived from bacteria. Therefore, it was unclear whether the data obtained by the analysis was suitable for the detection of *V. cholerae*. In order to examine whether the data shown in Fig. 2a can be used to clarify the infection status of *V. cholerae*, the ratio of the reads from *V. cholerae* to the reads of all bacteria in the sample was calculated (Fig. 2b). As a result, the reads from *V. cholerae* were detected even in samples with a low ratio of bacterial genes, as seen in patients 13, 25, and 29. Conversely, some patients, such as patients 10, 18, and 17, had a high proportion of bacterial genes but a low detection rate of the read from *V. cholerae* (Fig. 2a,b). From these results, we thought that the data obtained are useful for analyzing the infection status of *V. cholerae* in the intestinal tract of the examined patients. The data also showed that patient age did not affect the intestinal retention of *V. cholerae*.

**Figure 3:**
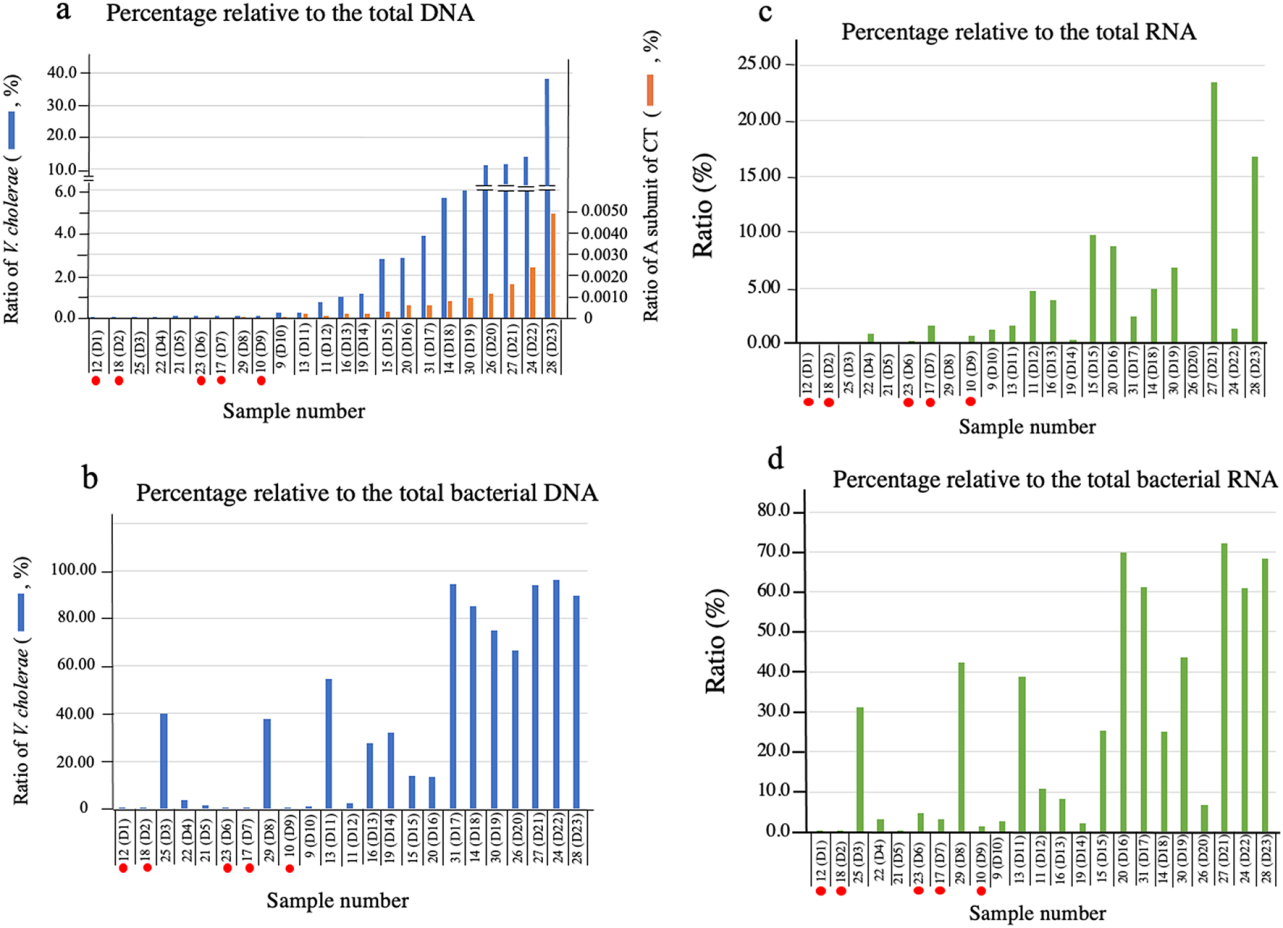
The ratios of DNA and RNA derived from *V. cholerae* in stool samples. The DNA and RNA in the stool samples from 23 patients who were diagnosed with cholera disease were extracted using a commercially available kit. Subsequently, the RNA samples were treated with DNase I to remove DNA from the samples. Reverse-transcribed DNA was prepared from these RNA samples using random primers and reverse transcriptase. The extracted DNA and reverse-transcribed DNA were investigated by a metagenomic sequencing analysis to clarify the origin of individual DNA and RNA. The origin of the DNA sequences was assigned by mapping to a database that included human and microorganism sequences. The obtained numbers of total reads, total bacterial reads, reads originating from *V. cholerae*, reads originating from *ctxA* in each sample are shown in Supplementary Tables S3 (the data from DNA) and S4 (the data from RNA). The percentages of reads of DNA from *V. cholerae* and from *ctxA* relative to the total reads are presented by blue bar and red bar in panel a, respectively. The percentages of reads of DNA from *V. cholerae* relative to the total bacterial reads are presented in panels b. Samples are arranged in ascending order of the ratio of reads from *V. cholerae* to the total reads in the DNA analysis. The ranking of each sample is presented by the numbers in parentheses starting with the letter D. The samples in these panels are arranged in the order of the D number. Similarly, the ratio of reads from *V. cholerae* to the total RNA reads and the total bacterial RNA are presented in panels c and d, respectively. The samples indicated by red circle are samples from the diarrheal stools of a patients who are focused on in this analysis.

In order to more clearly illustrate the presence of *V. cholerae* in the diarrheal stools of the patients examined, the ratio of reads from *V. cholerae* to total reads for each sample which was determined in Fig. 2a was sorted in descending order. The results are shown in Fig. 3a. The ratio (percentage) in each patient is indicated by the blue bar in the figure. The numbers in parentheses after the sample number, with D as the first letter, indicate the order from lowest to highest percentage obtained. As shown in Fig. 3a, the percentage of *V. cholerae* that the patients carried in their stools varied from 0.003% (sample 12(D1)) to 38.337% (sample 28(D23)).

However, what we want to reveal in this study is the presence of toxigenic *V. cholerae* producing CT. The genes presented by blue bar in Fig. 3a appear to contain the genes derived from toxigenic *V. cholerae*, but it cannot be concluded that they are. It is highly possible that other genes derived from such as *V. cholerae* not possessing *ctx* or bacteria having the same gene sequence as *V. cholerae,* are included. So, in order to examine the existence of *V. cholerae* possessing *ctx*, we examined the number of reads derived from *ctxA* (Supplementary Table S3). In the samples with D number 8 or more, the gene derived from *ctxA* was detected in all the samples except one sample (D9). The ratio of read from *ctxA* to the number of reads from total DNA is shown by the red bar in Fig. 3a. The ratio of the number of reads derived from *ctxA* to the total DNA was correlated with the ratio of the number of reads derived from the *V. cholerae* gene to the total DNA (Fig. 3a). From these results, it seems that most of the genes of *V. cholerae* detected in Fig. 3a are derived from *V. cholerae* possessing *ctx*.

Furthermore, the ratio of the number of reads of *V. cholerae* to the number of reads derived from total bacteria which was obtained in Fig. 2a, was arranged in the order used for the array in Fig. 3a (the order of the ratio of the number of reads from *V. cholerae* to the number of reads from total DNA) (Fig. 3b). From this arrangement of Fig. 3b, it can be seen that the sample with a large D head number has a large proportion of *V. cholerae* in the bacteria. The highest value was obtained from sample 24 (D22). The sample showed that 95.917% of the bacteria was *V. cholerae*.

On the other hand, in many samples with small D numbers, this ratio is small, but there are exceptions. For example, in samples 25 (D3), 29 (D8) and 13 (D11), the presence of *V. cholerae* is clear. Although not as clear as these three samples, the presence of *V. cholerae* in other samples such as 22 (D4), 21 (D5), 9 (D10) and 11(D12) is evident, although in small quantities (Fig. 3b). Therefore, it was considered that these patients were infected with *V. cholerae*. These results seem to accurately reflect the actual state of *V. cholerae* in the stool. Therefore, it was considered that the infection status of *V. cholerae* in the patient could be inferred from the obtained data.

As shown in Fig. 3b, in the samples of 18 (D2), 12 (D1), 17 (D7), 10 (D9)) and 23 (D6), the ratio of the read from *V. cholerae* to the read from total bacteria is very low at 0.032%, 0.118%, 0.225%, 0.244% and 0.285%, respectively. It was unknown whether these patients were infected with *V. cholerae* and developed diarrhea due to the infection with *V. cholerae*. Therefore, further examination was needed to determine if these patients were infected with *V. cholerae*. These five samples are marked by red circles in Fig. 3a,b.

Subsequently, we examined the ratio of the reads of RNA of *V. cholerae* to clarify the expression of the genes of *V. cholerae* in the intestinal lumen of these patients. RNA samples were prepared by different methods from the patient's stool and the RNA in these samples was analyzed by metagenomic sequencing analysis. The ratio of the number of reads derived from the RNA of *V. cholerae* to the number of reads derived from total RNA and to the number of reads derived from total bacterial RNA in the sample was determined. The results are shown in Fig. 3c,d, respectively. Samples that had fewer reads for genes derived from *V. cholerae* in the previous analysis of DNA reads (Fig. 3a,b)were also indicated with a red circle in Figs. 3c,d. These samples also had low amounts of RNA read from *V. cholerae*. In particular, the ratio of RNA read from *V. cholerae* to total bacterial RNA in samples 12 (D1) and 18 (D2) was low, 0.038% and 0.236%, respectively (Supplementary Table S4, Fig. 3d). Judging from these low values, it is doubtful that these two patients, patients 12 and 18, had diarrhea due to infection with *V. cholerae*.

#### Detection of *ctxA* by PCR

Subsequently, we amplified *ctxA* in the DNA samples extracted from the stool samples by PCR, in order to reconfirm the presence of *ctx* in stool samples. The PCR was performed using the same conditions used for the detection of *ctxA* in the isolates as described above in the “Sample collection and isolation of *V. cholerae* O1 possessing the CT gene” section of the “Results”. Amplification in this PCR was also done for 30 cycles.

**Figure 4:**
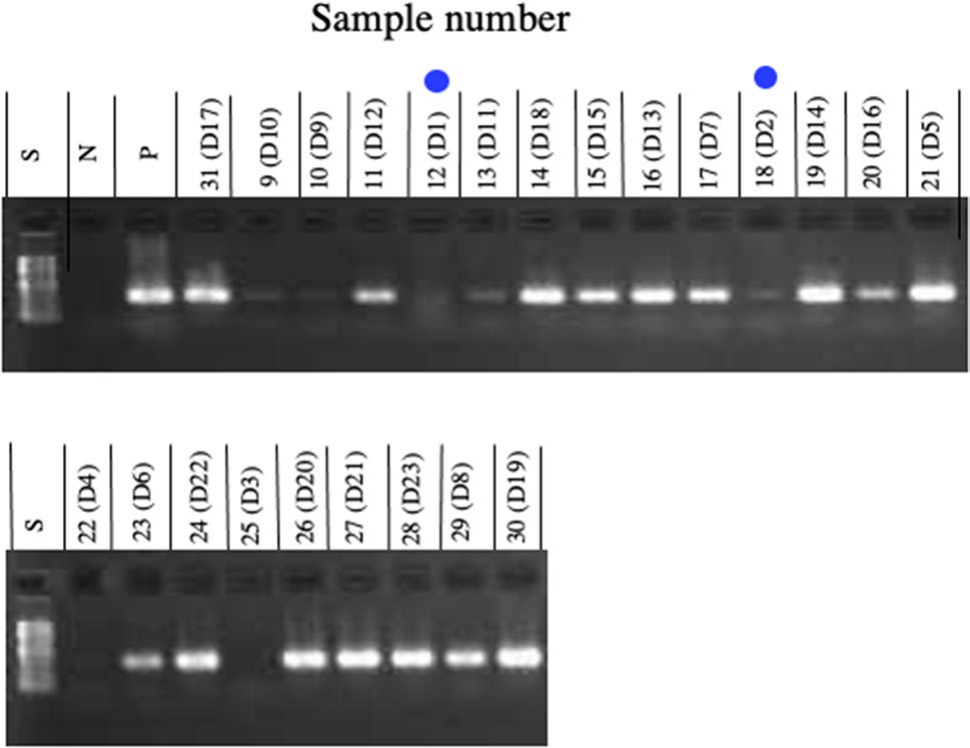
PCR to detect *ctxA* in the stool samples of diarrhea patients. DNA was extracted from the stool samples of 23 patients who were diagnosed with cholera disease. PCR to amplify *ctxA* in these DNA samples was performed using the specific primers *ctcagacgggatttgttaggcacg* and *tctatctctgtagcccctattacg*^6^, and the products were analyzed by agarose gel electrophoresis. The sample numbers are the same as the numbers shown in the footnotes of Fig. 3. Numbers beginning with D in parentheses show the order of the content of DNA from *V. cholerae* among these samples. The samples indicated by blue circle are samples from the diarrheal stools of patients (patients 12 and 18), who are focused on in this study. S: the size marker for gel electrophoresis; N: the negative control in which DNA was not added to the reaction mixture; P: the positive control in which DNA prepared from *V. cholerae* O1 N16961^28^ was added to the reaction mixture.

From the results of metagenomic sequencing shown in Fig. 3, we found that the samples from patient 12 (D1) and patient 18 (D2) contained few genes derived from *V. cholerae* O1. The results obtained by PCR are shown in Fig. 4. The samples from the two patients, 12 (D1) and 18 (D2), are indicated by blue circle. No distinct bands corresponding to *ctxA* were detected in the lanes analyzed sample 12(D1). Meanwhile, a very faint band was visible in the lane where the sample from 18(D2) was analyzed. However, it often happens that small amounts of sample are mixed into adjacent lanes when adding the sample to be analyzed in agar electrophoresis. Hence, we concluded that the amount of *ctxA* in these two samples amplified by PCR was very low. This supports our inference that the diarrhea in these two patients was not caused by the infection with *V. cholerae* O1.

Similarly, clear bands were not detected in samples 9(D10), 10(D9), 13(D11), 22(D4), and 25(D3). The results of metagenomic analysis of these samples showed that the number of read from *V. cholerae* was low and *ctxA* was either not detected (samples 10(D9), 22(D4) and 25(D3)) or was detected but in small amounts (samples 9(D10) and 13(D11) (Supplementary Table S3, Fig. 3a).

The amount of sample added to the reaction solution in the PCR reaction was as small as 5 µl, and it is not clear whether this small volume of solution contained the necessary amount of *ctxA* for the amplification in PCR. It is also possible that the sample contained substances that would inhibit amplification by PCR. For these reasons, we believe that no clear band corresponding to *ctxA* appeared in this PCR. However, it is clear from the results of Fig. 3b,d that these samples, (9(D10), 10(D9), 13(D11), 22(D4), and 25(D3)) contain the gene derived from *V. cholerae* (*ctx*). Therefore, we considered these four patients to be patients infected with *V. cholerae*.

#### The levels of CT and proteolytic activity in the stool samples

From the genetic studies in Figs. 3 and 4, it was inferred that *V. cholerae* O1 was not involved in the onset of the diarrhea in two patients (12(D1) and 18(D2)). However, this inference was based on amplification and analysis of genetic sample prepared from diarrhea stool of patients. There is no proof that the sample procurement and the analysis of sample was done reliably with high probability. Hence, we thought that it was necessary to analyze samples adjusted from different perspectives by different means.

Then, we challenged to measure the amount of CT. CT is the toxin responsible for the diarrhea caused by *V. cholerae* O1. CT is released into the intestinal lumen, where it acts on the intestinal cells of patients to induce diarrhea. Thus, we measured the CT content in the stool samples. In addition, we also measured the proteolytic activity in the stool samples, because CT is sensitive to proteolytic activity, and we were concerned that the CT would be degraded by proteases during storage outside of the body.

**Figure 5:**
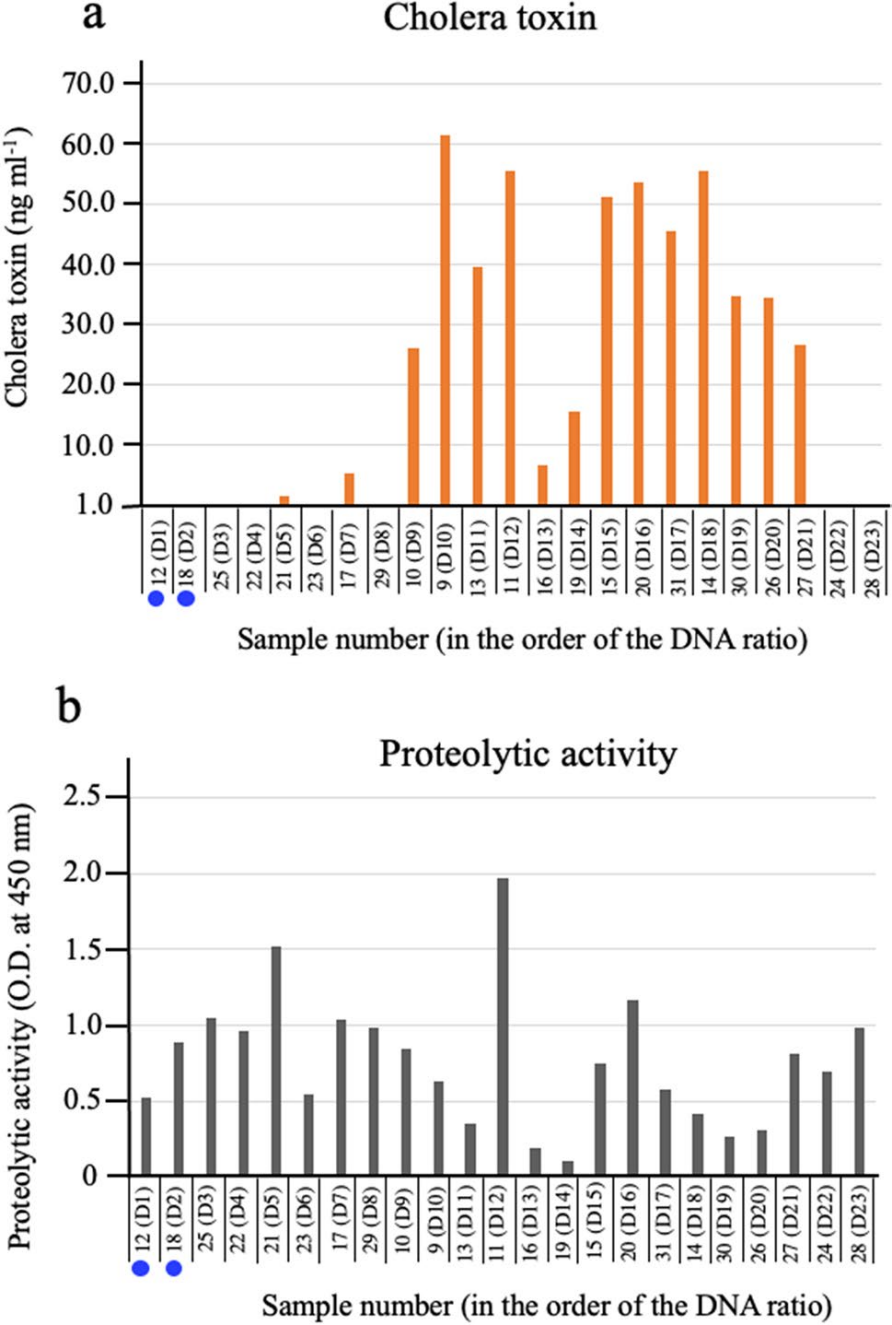
The levels of CT and proteolytic activity in the stool samples. Twenty-three stool samples of patients who were diagnosed with cholera disease were centrifuged at 10,000×*g* for 10 min. The CT content of the supernatants was determined using a GM1-ganglioside ELISA method^8^ (**a**). The proteolytic activity of the supernatants was determined by the lysis of casein^10^ (**b**). The sample numbers are the same as the numbers shown in the footnotes of Fig. 3. Numbers beginning with D in parentheses show the order of the content of DNA from *V. cholerae* among these samples. The samples in this figure are arranged in the order of the D numbers. A bar indicating the amount of CT is not drawn in the figure for the sample whose CT amount was below the detection limit. From the tests shown in Figs. 2, 3 and 4, samples of two patients who are unlikely to have diarrhea caused by the infection with *V. cholerae* are marked with a blue circle. O.D.: optical density.

The CT content and the proteolytic activity in the stool samples of the 23 cholera patients were measured by the GM1-ganglioside ELISA method and the lysis of casein, respectively^8,10^, and the results are presented in Fig. 5a,b, respectively.

Proteolytic activity was detected in all samples, although there were differences in the strengths of the activity. It was also found that high protease activity was not associated with decreased levels of CT in the samples, e.g., sample 11(D12) showed the highest protease activity among the samples examined, and the amount of CT in that sample was also high. Therefore, we considered that the proteolytic activity had almost no influence on the amount of CT in this study. Furthermore, the fact that protease activity was found in all samples indicated that these samples were collected and stored without any significant denaturation.

The ELISA method used in this assay can accurately detect CT at concentrations above 1.0 ng ml^−1^, but it is impossible to accurately determine the concentration of CT at concentrations below 1.0 ng ml^−1^. Therefore, we treated samples containing less than 1.0 ng ml^−1^ of CT as containing no CT.

As described above, we considered that the diarrhea in the two patients (12(D1) and 18(D2)) was not due to the infection with *V. cholerae* O1 from the genetic analysis. The analysis of CT in stool samples showed that the CT concentrations of these two samples were below the detection limit (Fig. 5a). This indicates that the number of *V. cholerae* O1 in the intestinal lumen of these patients, (12(D1) and 18(D2)), was extremely low at the time of sampling.

#### Investigation of diarrheagenic microorganisms in diarrheal stool

It was shown that diarrhea in patients 12 (D1) and 18 (D2) may have been caused by infection with microorganisms other than *V. cholerae*. Then we examined the data of metagenomic sequencing of these two patients to reveal the infected diarrhea-causing microorganisms (DDBJ Sequence Read Archive under the accession code PRJDB10675). As a result, we found that that DNA from the two bacteria, *Streptococcus pneumoniae* and *Salmonella enterica* was abundant in the stools of patients 12(D1) and 18(D2), respectively.

The ratios of DNA read of *St. pneumonia*e in DNA samples of patient 12(D1) to the total DNA and to the total bacterial DNA are 0.095% and 3.988%, respectively. These ratios of *V. cholerae* in this patient, 12 (D1), are 0.003% and 0.118%, respectively. And those of *S. enterica* in the stools of patients 18(D2) are 0.536% and 1.118%, respectively. And these ratios of *V. cholerae* in this patient, 18 (D2), are 0.015% and 0.032%, respectively (Supplementary Table 2).

These two bacteria, *St. pneumonia*e and *S. enterica,* are bacteria that are not detected as normal intestinal bacteria. As shown, these ratios of DNA of each bacteria in diarrheal stool are much higher than these of *V. cholerae*. Therefore, these two bacteria are considered to be related to these patients’ symptom, respectively.

Nonetheless, toxigenic *V. cholerae* O1 was also isolated from these two patients in laboratory bacteriology tests. It is likely that some of the very few *V. cholerae* O1 in the intestinal tract were extruded with the diarrhea and were subsequently detected by the enrichment culture for *V. cholerae*. This indicated that *V. cholerae* O1 may cause subclinical infections in residents of the Kolkata region of India. With this subclinical infection, the number of *V. cholerae* O1 inhabiting the intestinal tract might be small.

### Surveillance of patient samples where no diarrhea-causing microorganisms were detected

To detect people with a subclinical infection of *V. cholerae* O1, we further analyzed the specific-pathogen-free stool samples of diarrhea patients. “Specific-pathogen-free stool sample” refers to the stool samples in which no etiological agent of diarrhea, including *V. cholerae*, was detected by our bacterial examination in the laboratory.

The number of samples examined in this analysis was 22 (samples number 1001 to 1022). All 22 diarrhea patients examined were inpatients at ID hospital, Kolkata. From the 22 patients, 20 patient stool samples were collected on the 1st day of hospitalization, and the stools of the remaining two patients (patients 1004 and 2022) were collected on the 2nd day of hospitalization. Antibiotics were used in a limited manner in these patients. Ofloxacin was the only antibiotic administered, and only four patients (patients 1001, 1011, 1012, and 1021) were administered with it (Supplementary Table S1).

DNA and RNA were extracted from the stool samples, and the DNA and RNA were analyzed by a metagenomic sequencing analysis using the same method used in the analysis of diarrheal stools from cholera patients.

Reads of the genes from *V. cholerae* were detected in every sample, although the value varied from sample to sample (Supplementary Tables S5 and S6). Although reads of the genes from *V. cholerae* were detected in every sample, we do not believe that every stool sample examined contained *V. cholerae*. In the metagenomic analysis, if the base sequence of a read was common to multiple bacteria, the read was recognized as being derived from those multiple bacteria. Therefore, even if a bacterium is not present in the sample, the reads in common with other bacteria are counted as the reads of those bacteria, i.e., if a read from bacteria other than *V. cholerae* is homologous to a corresponding gene of *V. cholerae,* its detection indicates that one gene derived from *V. cholerae* was found in the sample. The total number of such reads is finally counted as the number of reads of *V. cholerae*. Therefore, it is unclear whether bacteria presenting a low read count are present in the sample. In order to solve these problems, not only the reads derived from *V. cholerae* but also the reads derived from *ctxA* were searched for in the sample.

**Figure 6:**
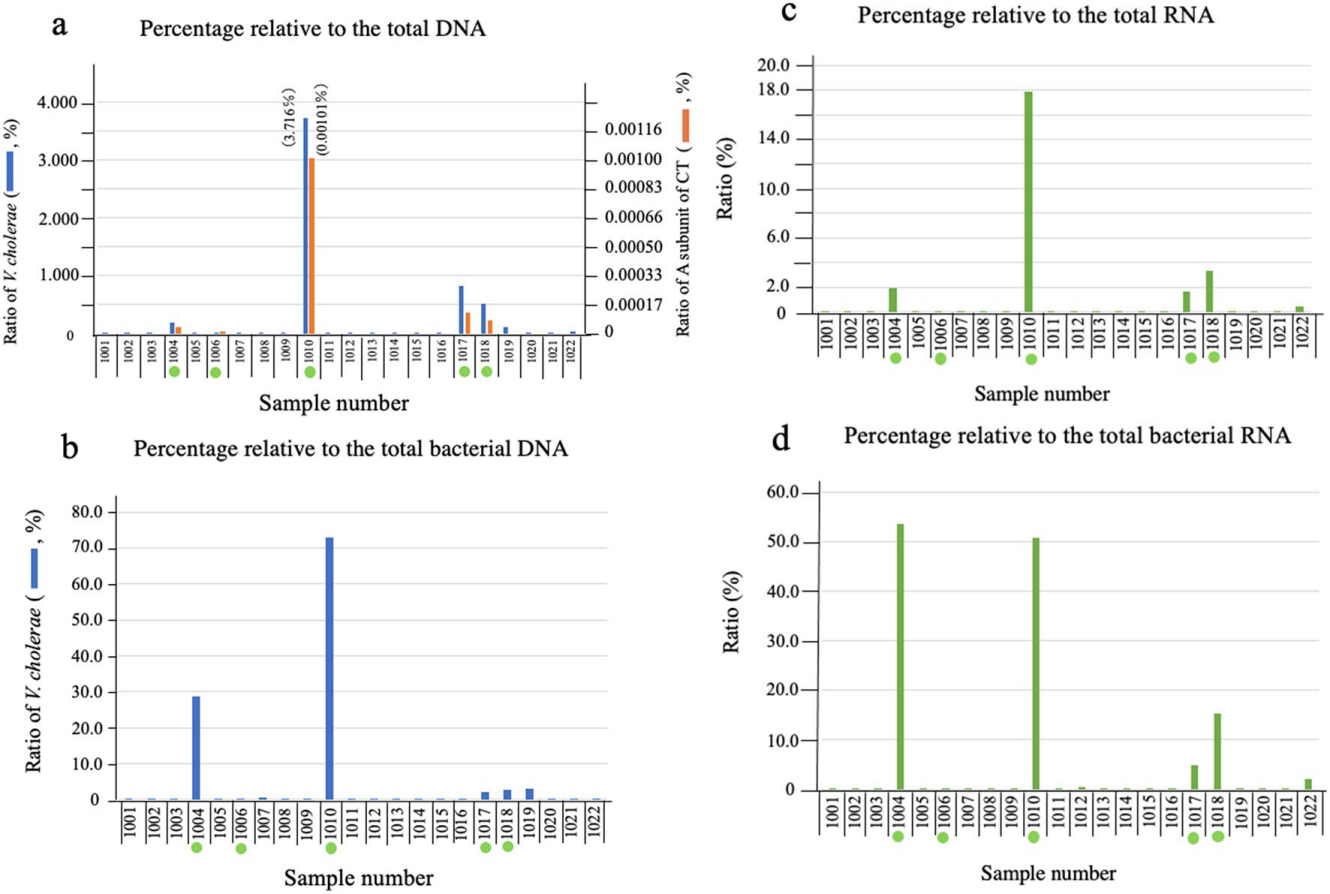
The ratio of DNA and RNA derived from *V. cholerae* in stool samples of the specific-pathogen-free patients. The stool samples from 22 diarrheal patients in which no etiological agent of diarrhea, including *V. cholerae*, was detected by our bacterial examination in the laboratory were analyzed in this examination. The extraction of DNA and RNA, and the preparation of reverse-transcribed DNA samples from the RNA samples were performed in the same manner as in Fig. 2. The origin of the reads obtained in this analysis was assigned by mapping to a database that included human and microorganism sequences. The obtained numbers of total reads, total bacterial reads, reads originating from *V. cholerae,* reads from *ctxA* in each sample are shown in Supplementary Tables S5 (the data from DNA) and S6 (the data from RNA). The percentages of reads of DNA from *V. cholerae* (blue bar in **a**) and of reads of DNA from *ctxA* (red bar in **a**) relative to the total DNA reads, and the percentages of reads of DNA from *V. cholerae* relative to the total bacterial DNA reads (**b**) are presented. Similarly, the results obtained from the RNA samples are presented in (**c**) and (**d**). The (**c**) and (**d**) show the percentages of reads of RNA from *V. cholerae* relative to the total RNA reads and to the reads of total bacterial RNA, respectively. The samples indicated by green circles are the samples of interest in this manuscript, as described in the text.

In addition, as described above, other DNA present in diarrheal stool, such as food-derived DNA, might hinder the analysis of the bacteria in the stool. As such, we determined four relative values of the number of reads from the genes of *V. cholerae*: the ratio of DNA reads of *V. cholerae* to the total DNA; the ratio of the DNA reads of *V. cholerae* to the total bacterial DNA; the ratio of the RNA reads of *V. cholerae* to the total RNA; and the ratio of the RNA reads of *V. cholerae* to the total bacterial RNA. Furthermore, we determined the relative value of the number of reads from *ctxA* to the total DNA (Supplementary Tables S5 and S6). These ratios are also shown in Fig. 6a–d.

The ratios of the number of reads derived from DNA of *V. cholerae* and the number of reads derived from *ctxA* to the number of reads of total DNA genes in these samples are shown by the blue and red bars in Fig. 6a, respectively. Reads from *ctxA* were detected in samples 1004, 1006, 1010, 1017 and 1018. This indicates that *V. cholerae* possessing *ctx* were alive in these samples; 1004, 1006, 1010, 1017 and 1018.

The ratio of *V. cholerae* to total bacterial DNA in these samples was examined. The results are shown in Fig. 6b. The proportion of DNA of *V. cholerae* to total bacteria DNA in the stool of patients 1004, 1006, 1010, 1017, and 1018 is 28.633%, 0.234%, 73.068%, 2.282%, and 2.774%, respectively (Fig. 6b).

In addition, the read of RNA from *V. cholerae* was examined. The ratio of the RNA to total RNA and to total bacterial RNA was calculated. RNA derived from *V. cholerae* was reliably detected in 4 of the 5 samples (1004, 1010, 1017, 1018). The ratio of the remaining one sample (1006) were low (Fig. 6c,d). However, it has been shown that the sample (1006) contains the read from DNA of *ctxA* (Supplementary Table S5). Therefore, we considered these five samples to be those containing toxigenic *V. cholerae*.

As antibiotics were not administered to these five patients, the effects of antibacterial agents could be disregarded in our examination of the bacterial species in the stools. Among these 5 samples, the ratio of samples 1004 and 1010 examined in this examination was high and comparable to those of the samples of the cholera patients (Figs. 3 and 6). We considered that the diarrhea of the patients 1004 and 1010, might have been caused by the infection with *V. cholerae* O1*.*

On the other hand, the samples of patients 1006, 1017 and 1018 did not show high values that could indicate that the diarrhea was caused by the infection with *V. cholerae*. It is probable that the diarrhea of these three patients (1006, 1017 and 1018) was caused by the actions of factors other than *V. cholerae* O1, and that a small number of *V. cholerae* inhabits the intestinal tract as a form of subclinical infection; this would explain why a gene derived from *V. cholerae* was detected by the metagenomic sequencing analysis. These results support the hypothesis that subclinical infections of *V. cholerae* occur in Kolkata.

## Discussion

We initiated the study to examine cholera patient stool by a metagenomic sequencing analysis to clarify the mode of infection of *V. cholerae* O1 and of other microorganisms in the diarrheal stool of cholera patients. However, the result from the metagenomic sequencing analysis showed that the stool of some cholera patients contained few genes derived from *V. cholerae* even though toxigenic *V. cholerae* O1 was isolated from the same stool samples. We thought that the number of *V. cholerae* O1 in the stool of these patients might be too small for *V. cholerae* O1 to be involved in the development of the diarrhea. So, to further investigate whether *V. cholerae* O1 is responsible for the diarrhea in these patients, we checked their stool for the *ctxA* gene by PCR, and we also measured the CT level in the stool. In addition, the number of reads derived from *ctxA* obtained by the metagenomic analysis was also checked. The stool of patients 12(D1) and 18(D2) was considered to be negative for *ctxA* and CT in these analyses. Therefore, it is unlikely that the diarrhea in these two patients (12(D1) and 18(D2) was caused by the infection with *V. cholerae* O1 in the intestinal tracts.

As described, toxigenic *V. cholerae* O1 was isolated from these patients by the usual bacteriological examination performed in the laboratory (Fig. 1). For these cases, the stool samples were cultured in alkaline peptone water (pH 8.5) to selectively grow *V. cholerae.* In such cultures, the number of *V. cholerae* O1 in samples increases dramatically, and *V. cholerae* O1 becomes easily detectable. Thus, even if the number of *V. cholerae* O1 in a sample is very small, *V. cholerae* O1 is easily isolated after selective culturing, leading to the diagnosis of cholera disease. However, this is a wrong diagnosis. To identify the true etiological agent and to make a correct diagnosis, a quantitative concept for the etiological agent must be introduced into the examination.

Then, we carefully searched the metagenomic analysis data of their stool samples of patients 12(D1) and 18(D2) to identify the true causative agents of their diarrhea including not only bacteria but also viruses and protozoans. Although no conclusive evidence was available to determine the causative organism of diarrhea of these patients, but interestingly, DNAs derived from *Streptococcus pneumoniae* and *Salmonella enterica* were detected in the stools of patients 12(D1) and 18(D2), respectively. As these two bacteria, *St. pneumonia*e and *S. enterica,* are bacteria that are not detected as normal intestinal bacteria, these two bacteria are considered to be related to these patients’ symptom, respectively.

*St. pneumoniae* is not usually isolated as a cause of diarrhea. However, recent studies of intestinal microbiota have reported that many *St. peumoniae* are present in the intestinal tract of infants with diarrhea^11^. Since patient 12 (D1) is 2 years old, it is possible that the diarrhea of patient 12(D1) was caused by the infection with *St. pneumoniae*. Additionally, patients with diarrhea caused by extraintestinal infection with *St. pneumoniae* have been reported^12^. Therefore, the relationship between the infection of *St. pneumoniae* and the onset of diarrhea is a problem to be solved in the future. On the other hand, it has been well known that *S. enterica* is an intestinal infectious agent that causes diarrhea^13^. Patient 18(D2) is believed to have had diarrhea due to the infection with *S. enterica.*

From these data, in Kolkata, we thought that that some residents in Kolkata may have a subclinical infection with *V. cholerae* O1. To get more information about the subclinical infection, we examined the stools of 22 diarrhea patients diagnosed as specific-pathogen-free. We found that the stools of 5 patients in the 22 patients contained the genes derived from *V. cholerae* possessing *ctxA*. From the number of the read of the gene, the number of *V. cholerae* inhabiting 3 samples (from patients 1006, 1017 and 1018) was considered to be small (Fig. 6). Therefore, *V. cholerae* appeared to be present in subclinical infections in these patients.

The main diarrhea-causing microorganisms of these 3 patients (patient numbers 1006, 1017, 1018) have not been fully investigated and are unknown. Metagenomic analysis of RNA samples recovered from these 3 samples showed that the RNA in sample 1006 contained a large amount of rotavirus A-derived RNA. Of the RNA in this sample, 36.933% was virus-derived RNA and 99.982% of the virus RNA was rotavirus A-derived RNA (PRJDB10675 (DDBJ/EMBL/GenBank accession number). This fact indicates that the diarrhea of this patient (patient 1006) was caused by rotavirus A infection. The number of *V. cholerae* in the stool of patient 1006 was a small. Therefore, it was unlikely that this patient's diarrhea was caused by an infection with *V. cholerae*. Inferring from these facts, the infection of *V. cholerae* in patient 1006 appeared to be a subclinical infection. This fact supports our hypothesis that subclinical infection of *V. cholerae* occurs in the inhabitants of Kolkata. The diarrhea-causing microorganisms of the other two patients (patients 1017, 1018) have remained unknown.

On the other hand, in order to accurately determine the causative microorganism of diarrhea, attention must be paid to the timing of stool collection. A sufficient number of causative microorganisms may not be present in the early or late stages of diarrhea. Nucleic acid derived from remnants of dead bacteria from past infections may also be contained in stool samples. Therefore, we think that it is important to track the microorganisms that cause diarrhea in a timely manner.

We collected almost diarrheal stools immediately after admission in this examination (Supplementary Table S1). The samples of diarrheal stool were taken during severe diarrhea. In addition, the patients who were followed up this time were not taking antibiotics. So, we thought that the microorganisms that caused the diarrhea in these patients were present in the stool samples at the time of collection of the stools. Therefore, we believe that the data on the diarrhea-causing microorganisms presented in this manuscript provide accurate information on the diarrhea-causing microorganisms of these patients.

If the people who have a small number of *V. cholerae* O1 in their intestines do not develop diarrhea, they are healthy carriers of *V. cholerae* O1. The existence of cholera carriers was reported in the 1960s in Kolkata, the Philippines, and east Pakistan^14–18^, although research on the carriers had been stagnant since then. Recently, however, the reports have been published reaffirming the subclinical infection of *V. cholerae* O1^19,20^. Our data might be the additional approval of this subclinical infection of *V. cholerae* using a genetic analysis technique.

Cholera patients emerge throughout the year in the Kolkata area, although the number of patients is small during December and January. In addition, the emergence of cholera patients is sporadic, and there have been no collective outbreaks in certain areas with cholerae disease in the Kolkata area^1^. This type of sporadic occurrence indicates that the source of infection of cholera disease is widely scattered in the Kolkata area. From these observations, we worry about the possibility that in the Kolkata area, the people who possess small numbers of *V. cholerae* 01 in their gut may be a source of cholera disease in this area.

The involvement of human carriers in the spread of infection by some enteric bacteria, such as *Salmonella*^21^, *Shigella*^22–24^, and diarrheagenic *E. coli*^25,26^, has been reported. In addition, the possibility of the involvement of human carriers in the spread of infection by *Campylobacter* has been proposed^27^.

Our study has shown that human carriers may be involved in the spread of infection with *V. cholerae*. Further studies will reveal the exact role of human carriers of *V. cholerae* and will contribute to the eradication of cholera disease.

## Methods

### Ethics approval

The examination carried out in India were approved by two committees, the Institutional Ethical Committee (IEC) of ICMR- National Institute of Cholera and Enteric Diseases (NICED), Kolkata, India (approved no. A-1/2015-IEC), and the Ethics Committee of Okayama University (approved no. Eki 90). The analysis of the DNA and RNA performed in Japan were approved by two committees of different organizations in Japan prior to initiation, the Ethics Committee of Okayama University (approved no. Eki 90) and the Ethics Committee of Osaka University (approved no. 583). The operation was carried out in accordance with relevant guidelines and regulations of the approval of these committees.

Informed consent was obtained from each adult patient or the parent/guardian of each child patient enrolled in this study.

### Sample collection and isolation of *V. cholerae* O1 possessing *ctx*

Forty-five stool samples were collected from diarrhea patients hospitalized at Beliaghata ID Hospital in Kolkata, India, between August 2017 and September 2018. Subsequently, the etiological examinations for diarrhea in these stools were carried out. The approval for these operations has been obtained from two ethics committee, Ethics Committee of NICED and Ethics Committee of Okayama University. The approval numbers are A-1/2015-IEC and Eki 90, respectively. The operations were performed in accordance with relevant guidelines and regulations of the two committees.

Forty-one of the stool samples were collected from the patients on the first day of hospitalization, three samples were collected on the second day of hospitalization, and one sample was collected on the fourth day of hospitalization (Supplementary Table S1). More than 10 g per sample were collected. The bacteriological examination was performed using a portion of each sample, and the remaining portion of each sample was stored at − 80 °C until use.

Etiological examinations in the laboratory were carried out as described previously^1^ and more than 17 kinds of diarrheagenic agents were tested for. For the detection of *V. cholerae* O1, the stool samples were cultured in alkaline peptone water (L-broth, pH 8.5; Becton, Dickinson and Company, Sparks, MD, USA) overnight at 37 °C, then the cultures were inoculated onto thiosulfate citrate bile-salt sucrose agar (Eiken Co., Tokyo, Japan), and incubated at 37 °C for 15 h. Sucrose-fermenting yellow colonies were examined for the oxidase reaction using *N,N,N’,N’*-tetramethyl-*p*-phenylenediamine (Sigma-Aldrich, St. Louis, MO, USA) as a substrate, and the oxidase-positive colonies were then inoculated into triple sugar iron agar slants, and cultured at 37 °C for 15 h. The isolates showing the characteristics of acid (+), gas (−), and H_2_S (−) were selected. O1 and O139 serogroup determination of the isolates was performed using a slide agglutination test with commercially available antisera (Denkaseiken Co., Ltd., Tokyo, Japan). The isolates showing a positive reaction with O1 antiserum in the agglutination test were classified as *V. cholerae* O1. Among the samples examined in this study, there were no isolates that reacted with O139 antiserum.

The presence of *ctx* in *V. cholerae* O1 was examined by PCR with primers designed to detect *ctxA* (forward primer: 5′-ctcagacgggatttgttaggcacg-3′, reverse primer: 5′-tctatctctgtagcccctattacg-3′). The expected length of the PCR product from these primers was 302 bp (NCBI GenBank nucleotide sequence database, accession number NC_002505 and NC_002506)^28^.

DNA samples of *V. cholerae* O1 were extracted using a heating method. The bacteria cultured in 2 ml of L-broth were collected by centrifugation, and suspended in 0.5 ml of distilled water. The samples were heated in boiling water for 10 min, then centrifuged. The obtained supernatant from each isolate was used as a DNA sample for PCR, and the PCR products were examined by agarose gel electrophoresis.

### Measurement of the CT level and proteolytic activity in the samples

The level of CT in samples was measured using a GM1-ganglioside ELISA method^8^. The proteolytic activity of the stool samples was determined using the lysis of casein^10^. The approval for these operations has been obtained from two ethics committee, Ethics Committee of NICED and Ethics Committee of Okayama University. The approval numbers are A-1/2015-IEC and Eki 90, respectively. The operations were performed in accordance with relevant guidelines and regulations of the two committees.

To measure the level of secreted CT from *V. cholerae* O1 in the culture medium, isolates were cultured statically in AKI medium^7^ at 37 °C for 24 h. The cultures were centrifuged (12,000×*g* for 15 min), and the supernatants were recovered. The CT content of these supernatants was measured using the GM1-ganglioside ELISA method as follows. Samples were diluted twofold or more with phosphate-buffered saline (PBS) before being used for measurements.

A 96-well microplate was coated with monosialoganglioside-GM1 (Sigma-Aldrich; 1 µg/ml in PBS containing 60 mM Na_2_CO_3_) by incubation at 37 °C for 4 h. Then, the plate was washed three times with PBS containing 0.05% Tween-20 (PBST). For blocking, the wells were incubated with 2% bovine serum albumin in PBS containing 60 mM Na_2_CO_3_ at 4 °C overnight. After washing the wells three times with PBST, 100 µl of the samples, which had been adequately diluted with PBS, was applied to the wells, and incubated for 2 h at room temperature. In addition, purified CT (Sigma-Aldrich) was serially diluted (0.625 to 10.0 ng/ml) and used in the same manner to prepare a standard curve. After washing the wells three times with PBST, the samples were incubated with a 1:20,000 dilution of rabbit anti-CT antibody (Sigma-Aldrich) for 1 h at room temperature. Then, they were washed three times with PBST, and reacted with a 1:2500 dilution of anti-rabbit horseradish peroxidase-conjugated antibody (Abcam, Cambridge, UK) for 1 h at room temperature, followed by three washes with PBST. Subsequently, 100 µl of the chromogenic tetramethylbenzidine substrate (Becton Dickinson Biosciences, Franklin Lakes, NJ, USA) was applied to the wells, and the plate was kept at room temperature for a few minutes until the color appeared. Then, the reaction was stopped by the addition of an equal volume of 3 M H_2_SO_4_, and the optical density was measured at 450 nm. The concentration of CT in each sample was determined using the standard curve obtained from the measurements of the serially diluted purified CT.

To measure the level of CT in stool samples, the stool samples were centrifuged at 10,000×*g* for 10 min, and the supernatants were recovered. The CT content of the supernatants was measured using the GM1-ganglioside ELISA method as described above.

To measure proteolytic activity, the stool samples were centrifuged at 10,000×*g* for 10 min, and the supernatants were recovered. A portion (40 µl) of the supernatant was added to a reaction mixture containing 0.4% azocasein and 10 mM Tris–HCl buffer (pH 7.5) in a final volume of 100 µl. The mixture was incubated at 37 °C for 1 h, and the reaction was stopped by adding 100 µl of 10% trichloroacetic acid. After centrifugation at 13,000×*g*, 100 µl of the supernatant was mixed with an equal volume of 1 N NaOH, and the optical density of the solution was measured at 450 nm.

### Extraction of DNA and RNA from stool samples and analysis by metagenomic shotgun sequencing

To analyze the nucleic acids (DNA and RNA) in these stool samples, these nucleic acids were extracted and analyzed by metagenomic sequencing. The approval for these operations has been obtained from two ethics committee, Ethics Committee of Okayama University and Ethics Committee of Osaka University. The approval numbers are Eki 90 and 583, respectively.

In the extraction of DNA and RNA from diarrheal stool samples, the fluid fraction recovered from the stool samples of patients was used as the raw material. Extraction of DNA and RNA from stool samples was performed using a DNeasy PowerSoil DNA Kit (Qiagen, Hilden, Germany) and a QIAamp MinElute Virus Spin Kit (Qiagen), respectively. Two hundred microliters of the fluid was used for each extraction, which was performed according to the manufacturer’s instructions. The extracted DNA and RNA were suspended in 100 µl of the designated buffer in each kit.

The concentrations of the obtained DNA and RNA were measured using an ultraviolet absorption measurement method. The values obtained from the samples of cholera patients are shown in Supplementary Tables S3 and S4.

For metagenomic shotgun sequencing, the RNA samples were treated with DNase I (Takara Bio Inc., Shiga, Japan) to remove DNA, then used to prepare reverse-transcribed DNA. Reverse transcription using SuperScript III reverse transcriptase (Invitrogen, Carlsbad, CA, USA) and N9 Random Primer (nonadeoxyribonucleotide mixture; pd (N)_9_; Takara Bio Inc.) was carried out to generate reverse-transcribed DNA.

Metagenomic shotgun libraries were constructed from both the DNA samples and the reverse-transcribed DNA samples using a Nextera XT DNA Library Prep Kit (Illumina, San Diego, CA, USA).

One hundred-base pair single-end sequencing was performed using a HiSeq 2500 (Illumina). Raw read quality was checked by FastQC (v0.11.3). We confirmed that all bases of all reads were Q28 or higher, and that they were Q30 or higher after adapter trimming. The adapter trimming was performed using cutadapt v1.9.2, and the number obtained after the trimming was regarded as the number of read after quality filtering. The minimum read size after quality trimming is 30.

After the trimming, sequence reads were mapped onto the database of iMetDB (http://imet.gen-info.osaka-u.ac.jp/en/imetdb.html), which includes human and microorganism sequences from NCBI-nt, by the Burrows-Wheeler Alignment tool (BWA) with default parameters^9^. The taxonomic information of each sequence was assigned and summarized by text mining of the mapping output files using BioRuby.

The read derived from ctxA was detected using the sequence from 1,567,338 to 1,568,114 of *V. cholerae* O1 biovar eltor str. N16961 chromosome I (GenBank: AAF94614.1).

## Supplementary Information

Supplementary Tables.

## Data Availability

The raw sequencing data have been deposited into the DDBJ Sequence Read Archive under the accession code PRJDB10675 (DDBJ/EMBL/GenBank accession number).
