# Supplementary material for: Metagenomic analysis of diarrheal stools in Kolkata, India, indicates the possibility of subclinical infection of Vibrio cholerae O1
Source: Sci Rep. 2022 Nov 14;12:19473. doi: 10.1038/s41598-022-24167-9 (PMC9663449; doi:10.1038/s41598-022-24167-9)
Supplement: Supplementary file 1 — Supplementary Tables. [file 41598_2022_24167_MOESM1_ESM.docx]

**Supplementary information**

# Supplementary Table S1, The age of patient, date of hospital admission, stool sampling date, and medicines administered to the patients.

# Supplementary Table S2, Data from the metagenomic sequencing analysis of DNA sequences published in the NIH Human Microbiome Project.

# Supplementary Table S3, Data from the metagenomic sequencing analysis of DNA samples extracted from the stools of 23 diarrheal patients diagnosed with cholera.

# Supplementary Table S4, Data from the metagenomic sequencing analysis of RNA samples extracted from the stools of 23 diarrheal patients diagnosed with cholera.

# Supplementary Table S5, Data from the metagenomic sequencing analysis of DNA samples extracted from the stools of 22 diarrheal patients in which the etiological agent of the diarrhea was not detected.

# Supplementary Table S6, Data from the metagenomic sequencing analysis of RNA samples extracted from the stools of 22 diarrheal patients in which the etiological agent of the diarrhea was not detected.

# Supplementary Table S1 | The age of patient, date of hospital admission, stool sampling date, and medicines administered to the patients.

| Patient  number | Age of patient | Admission date | Sampling date | Pathogen　isolated^a^ | Medicines administered |
| --- | --- | --- | --- | --- | --- |
| 9 | 22 | May 2nd, 2018 | May 2nd, 2018 | *V. cholerae* O1 | Probiotics, Ondansetron |
| 10 | 19 | May 2nd, 2018 | May 5th, 2018 | *V. cholerae* O1 | Probiotics, Ondansetron, Ranitidine |
| 11 | 41 | May 2nd, 2018 | May 2nd, 2018 | *V. cholerae* O1 | Probiotics, Ondansetron, Ranitidine |
| 12 | 2 | May 2nd, 2018 | May 2nd, 2018 | *V. cholerae* O1 | Probiotics, Ondansetron, Zinc |
| 13 | 12 | May 4th, 2018 | May 4th, 2018 | *V. cholerae* O1 | Probiotics, Ondansetron |
| 14 | 15 | May 4th, 2018 | May 4th, 2018 | *V. cholerae* O1 | Probiotics, Ondansetron |
| 15 | 52 | May 4th, 2018 | May 4th, 2018 | *V. cholerae* O1 | Probiotics, Ondansetron, Ranitidine |
| 16 | 17 | May 7th, 2018 | May 7th, 2018 | *V. cholerae* O1 | Probiotics |
| 17 | 32 | May 9th, 2018 | May 9th, 2018 | *V. cholerae* O1 | Probiotics, Ondansetron, Ranitidine, Dicyclomine |
| 18 | 24 | May 9th, 2018 | May 9th, 2018 | *V. cholerae* O1 | Probiotics, Ondansetron, Dicyclomine |
| 19 | 40 | May 9th, 2018 | May 9th, 2018 | *V. cholerae* O1 | Probiotics |
| 20 | 33 | May 11th, 2018 | May 11th, 2018 | *V. cholerae* O1 | Probiotics, Ondansetron, Ranitidine |
| 21 | 25 | May 11th, 2018 | May 11th, 2018 | *V. cholerae* O1 | Probiotics, Ondansetron, Ranitidine |
| 22 | 58 | May 16th, 2018 | May 16th, 2018 | *V. cholerae* O1 | Probiotics, Ondansetron, Ranitidine, Dicyclomine |
| 23 | 40 | July 2nd, 2018 | July 2nd, 2018 | *V. cholerae* O1 | Probiotics, Ondansetron, Ranitidine, Dicyclomine |
| 24 | 22 | July 2nd, 2018 | July 2nd, 2018 | *V. cholerae* O1 | Probiotics, Ondansetron, Ranitidine, Dicyclomine |
| 25 | 65 | August 8th, 2018 | August 8th, 2018 | *V. cholerae* O1 | Probiotics, Ondansetron, Ranitidine, Dicyclomine |
| 26 | 16 | August 8th, 2018 | August 8th, 2018 | *V. cholerae* O1 | Probiotics, Ondansetron, Ranitidine |
| 27 | 30 | August 29th, 2018 | August 29th, 2018 | *V. cholerae* O1 | Probiotics, Ondansetron, Ranitidine |
| 28 | 35 | September 3rd, 2018 | September 3rd, 2018 | *V. cholerae* O1 | Probiotics, Ondansetron, Ranitidine, Zinc |
| 29 | 65 | September 10th, 2018 | September 11th, 2018 | *V. cholerae* O1 | Probiotics, Ondansetron, Ranitidine, Zinc |
| 30 | 50 | September 14th, 2018 | September 14th, 2018 | *V. cholerae* O1 | Probiotics, Ondansetron, Ranitidine, Zinc |
| 31 | 19 | May 2nd, 2018 | May 2nd, 2018 | *V. cholerae* O1 | Probiotics, Ornidazole, Ranitidine, Dicyclomine |
| 1001 | 19 | August 21st, 2017 | August 21st, 2017 | Specific-pathogen-free^b^ | Ofloxacin, Probiotics, Ondansetron, Ranitidine, Ornidazole |
| 1002 | 41 | September 11th, 2017 | September 11th, 2017 | Specific-pathogen-free | Probiotics, Paracetamol, Ondansetron |
| 1003 | 30 | October 3rd, 2017 | October 3rd, 2017 | Specific-pathogen-free | Probiotics, Paracetamol, Ondansetron, Ranitidine |
| 1004 | 1 | October 25th, 2017 | October 26th, 2017 | Specific-pathogen-free | Probiotics, Ondansetron, Ranitidine, Zinc |
| 1005 | 52 | November 1st, 2017 | November 1st, 2017 | Specific-pathogen-free | Probiotics, Ondansetron, Ranitidine, Dicyclomine, Paracetamol |
| 1006 | 0.1 | November 1st, 2017 | November 1st, 2017 | Specific-pathogen-free | Probiotics, Paracetamol |
| 1007 | 50 | November 6th, 2017 | November 6th, 2017 | Specific-pathogen-free | Probiotics, Paracetamol, Pantoprazole |
| 1008 | 30 | November 7th, 2017 | November 7th, 2017 | Specific-pathogen-free | Probiotics, Paracetamol, Ondansetron |
| 1009 | 16 | November 8th, 2017 | November 8th, 2017 | Specific-pathogen-free | Probiotics, Paracetamol, Dicyclomin |
| 1010 | 1 | November 9th, 2017 | November 9th, 2017 | Specific-pathogen-free | Probiotics, Paracetamol, Ondansetron |
| 1011 | 54 | August 30th, 2017 | August 30th, 2017 | Specific-pathogen-free | Ofloxacin, Probiotics, Ondansetron, Ranitidine, Ornidazole |
| 1012 | 36 | August 30th, 2017 | August 30th, 2017 | Specific-pathogen-free | Ofloxacin, Probiotics, Paracetamol, Ranitidine, Ornidazole |
| 1013 | 80 | September 11th, 2017 | September 11th, 2017 | Specific-pathogen-free | Probiotics, Paracetamol, Ondansetron |
| 1014 | 65 | October 3rd, 2017 | October 3rd, 2017 | Specific-pathogen-free | Probiotics, Paracetamol, Ondansetron |
| 1015 | 70 | October 3rd, 2017 | October 3rd, 2017 | Specific-pathogen-free | Probiotics, Paracetamol, Ondansetron |
| 1016 | 35 | October 4th, 2017 | October 4th, 2017 | Specific-pathogen-free | Probiotics, Ornidazole, Ranitidine, Dicyclomine |
| 1017 | 62 | October 4th, 2017 | October 4th, 2017 | Specific-pathogen-free | Probiotics, Ornidazole |
| 1018 | 60 | October 30th, 2017 | October 30th, 2017 | Specific-pathogen-free | Probiotics |
| 1019 | 23 | October 30th, 2017 | October 30th, 2017 | Specific-pathogen-free | Probiotics, Ornidazole |
| 1020 | 1 | November 1st, 2017 | November 1st, 2017 | Specific-pathogen-free | Probiotics, Ornidazole |
| 1021 | 22 | August 30th, 2017 | August 30th, 2017 | Specific-pathogen-free | Ofloxacin, Probiotics, Ondansetron, Ranitidine, Ornidazole |
| 1022 | 4 | August 31st, 2017 | September 1st, 2017 | Specific-pathogen-free | Probiotics, Ondansetron, Zinc |

^a^Detection of pathogenic microorganisms in the stool was performed using the method reported previously in the laboratory^1^.

^b^“Specific-pathogen-free” indicates that pathogenic microorganisms were not detected in microbiological tests performed in the laboratory.

# Supplementary Table S2| Number of read obtained by the metagenomic sequencing analysis of DNA sequences published in the NIH Human Microbiome Project.

| Code^a^ | Sex^b^ | Total read | Read obtained after quality filtering^c^ | Total read from bacteria | Read from *V. cholerae* |
| --- | --- | --- | --- | --- | --- |
| SRS014459 | Female | 40,946,097 | 40,920,606 | 11,027,457 | 0 |
| SRS014613 | Female | 52,872,663 | 52,856,010 | 13,954,562 | 8 |
| SRS011405 | Female | 61,020,717 | 60,975,797 | 17,837,420 | 12 |
| SRS022524 | Female | 10,372,511 | 10,301,809 | 3,036,614 | 0 |
| SRS022713 | Female | 43,777,373 | 43,756,471 | 9,038,072 | 0 |
| SRS024388 | Female | 59,842,162 | 59,825,098 | 18,849,883 | 2 |
| SRS054590 | Female | 43,016,703 | 42,970,794 | 13,488,682 | 0 |
| SRS064276 | Female | 43,579,625 | 43,549,062 | 10,478,972 | 10 |
| SRS1055043 | Female | 24,095,763 | 24,032,052 | 6,056,359 | 4 |
| SRS893172 | Female | 11,843,941 | 11,825,500 | 4,248,734 | 0 |
| SRS013158 | Male | 55,463,878 | 55,434,510 | 17,181,885 | 0 |
| SRS017247 | Male | 43,538,871 | 43,509,550 | 10,990,494 | 11 |
| SRS018313 | Male | 54,440,527 | 54,409,466 | 25,701,207 | 6 |
| SRS018575 | Male | 46,551,042 | 46,371,988 | 12,310,332 | 3 |
| SRS045645 | Male | 53,212,622 | 53,186,709 | 17,761,823 | 0 |
| SRS047014 | Male | 45,016,391 | 44,950,676 | 13,199,979 | 2 |
| SRS049164 | Male | 28,931,340 | 28,903,628 | 7,990,729 | 2 |
| SRS064645 | Male | 11,083,351 | 11,002,113 | 2,256,822 | 2 |
| SRS104975 | Male | 28,192,689 | 28,048,657 | 9,111,198 | 7 |
| SRS017191 | Male | 50,425,827 | 50,401,621 | 14,795,699 | 11 |

^a^Code of each file published in the NIH Human Microbiome Project (https://www.hmpdacc.org/hmp/hmp/hmasm2/).

^b^Gender of the contributor of each sample.

^c^Number of the read after quality filtering was obtained by the trimming.

Supplementary Table S3 | Data from the metagenomic sequencing analysis of DNA samples extracted from the stools of 23 diarrheal patients diagnosed with cholera.

| DNA sample | | | | | | | | | | | |
| --- | --- | --- | --- | --- | --- | --- | --- | --- | --- | --- | --- |
| Sample No. | Conc. of sample (ng/µl)^a^ | Total read | Read obtained after quality filtering^b^ | Total DNA read from bacteria | Ratio of read from bacteria per read after quality filtering (%) | DNA read from *V. cholerae* | DNA read from *ctxA*^c^ | Ratio of read from *V. cholerae* to total DNA read (%)^d^ | Ratio of read from *V. cholerae* to read obtained after quality filtering (%) | Ratio of read from *V. cholerae* to bacterial DNA read (%)^e^ | Ratio of read from *ctxA* to total DNA read (%)^f^ |
| 9 | 0.55 | 4,898,389 | 4,897,818 | 1,204,733 | 24.597 | 11,427 | 1 | 0.233 | 0.233 | 0.949 | 0.00002 |
| 10 | 0.80 | 3,946,394 | 3,945,611 | 1,832,956 | 46.456 | 4,476 | 0 | 0.113 | 0.113 | 0.244 | 0.00000 |
| 11 | 0.40 | 4,876,580 | 4,875,577 | 1,587,155 | 32.553 | 35,110 | 4 | 0.720 | 0.720 | 2.212 | 0.00008 |
| 12 | 11.95 | 6,456,875 | 6,446,410 | 153,426 | 2.380 | 181 | 0 | 0.003 | 0.003 | 0.118 | 0.00000 |
| 13 | 0.15 | 6,258,014 | 6,255,270 | 27,726 | 0.443 | 15,061 | 11 | 0.241 | 0.241 | 54.321 | 0.00018 |
| 14 | 1.75 | 5,488,560 | 5,480,391 | 363,604 | 6.635 | 310,013 | 43 | 5.648 | 5.657 | 85.261 | 0.00078 |
| 15 | 0.65 | 5,335,138 | 5,333,146 | 1,056,250 | 19.805 | 147,466 | 15 | 2.764 | 2.765 | 13.961 | 0.00028 |
| 16 | 0.85 | 5,309,560 | 5,309,205 | 191,512 | 3.607 | 52,507 | 10 | 0.989 | 0.989 | 27.417 | 0.00019 |
| 17 | 1.45 | 3,651,309 | 3,649,820 | 1,717,776 | 47.065 | 3,863 | 0 | 0.106 | 0.106 | 0.225 | 0.00000 |
| 18 | 2.20 | 3,626,009 | 3,625,280 | 1,737,731 | 47.934 | 559 | 0 | 0.015 | 0.015 | 0.032 | 0.00000 |
| 19 | 2.65 | 5,264,892 | 5,264,357 | 183,748 | 3.490 | 58,675 | 10 | 1.114 | 1.115 | 31.932 | 0.00019 |
| 20 | 1.60 | 5,299,381 | 5,295,058 | 1,091,782 | 20.619 | 147,828 | 30 | 2.790 | 2.792 | 13.540 | 0.00057 |
| 21 | 10.30 | 5,763,549 | 5,753,412 | 345,214 | 6.000 | 4,393 | 0 | 0.076 | 0.076 | 1.273 | 0.00000 |
| 22 | 1.95 | 5,771,445 | 5,761,975 | 68,398 | 1.187 | 2,418 | 0 | 0.042 | 0.042 | 3.535 | 0.00000 |
| 23 | 3.30 | 4,213,114 | 4,212,941 | 1,195,755 | 28.383 | 3,406 | 0 | 0.081 | 0.081 | 0.285 | 0.00000 |
| 24 | 3.60 | 5,733,286 | 5,728,375 | 841,772 | 14.695 | 807,403 | 136 | 14.083 | 14.095 | 95.917 | 0.00237 |
| 25 | 1.35 | 6,661,307 | 6,657,563 | 3,528 | 0.053 | 1,408 | 0 | 0.021 | 0.021 | 39.909 | 0.00000 |
| 26 | 5.05 | 4,963,373 | 4,963,171 | 839,455 | 16.914 | 556,663 | 57 | 11.215 | 11.216 | 66.312 | 0.00115 |
| 27 | 3.60 | 5,327,104 | 5,318,232 | 643,938 | 12.108 | 605,728 | 83 | 11.371 | 11.390 | 94.066 | 0.00156 |
| 28 | 1.85 | 5,775,614 | 5,774,575 | 2,477,967 | 42.912 | 2,214,194 | 283 | 38.337 | 38.344 | 89.355 | 0.00490 |
| 29 | 3.75 | 6,055,177 | 6,044,847 | 17,081 | 0.283 | 6,447 | 1 | 0.106 | 0.107 | 37.744 | 0.00002 |
| 30 | 7.50 | 5,036,118 | 5,031,223 | 402,926 | 8.009 | 301,769 | 46 | 5.992 | 5.998 | 74.894 | 0.00091 |
| 31 | 22.50 | 5,869,338 | 5,859,506 | 238,152 | 4.064 | 224,666 | 34 | 3.828 | 3.834 | 94.337 | 0.00058 |

^a^Concentration of DNA in the samples.

^b^Number of the read after quality filtering was obtained by the trimming.

^c^*ctxA*; the gene for A subunit of cholera toxin.

^d^Percentage of reads from *V. cholerae* relative to the total reads.

^e^Percentage of reads from *V. cholerae* relative to the total bacterial reads.

^f^Percentage of reads from *ctxA* relative to the total reads.

Supplementary Table S4 | Data from the metagenomic sequencing analysis of RNA samples extracted from the stools of 23 diarrheal patients diagnosed with cholera.

| RNA sample | | | | | | |
| --- | --- | --- | --- | --- | --- | --- |
| Sample No. | Conc. of sample (ng/µl)^a^ | Total reads | Total RNA reads from bacteria | Reads from *V. cholerae* | Ratio to the total RNA (%)^b^ | Ratio to the total bacterial RNA (%)^c^ |
| 9 | 2.44 | 5,057,043 | 2,522,745 | 66,184 | 1.309 | 2.623 |
| 10 | 2.08 | 4,102,066 | 2,053,975 | 29,978 | 0.731 | 1.460 |
| 11 | 6.56 | 5,836,233 | 2,558,683 | 277,178 | 4.749 | 10.833 |
| 12 | 9.80 | 4,582,056 | 2,211,209 | 843 | 0.018 | 0.038 |
| 13 | 5.24 | 3,074,621 | 128,939 | 49,894 | 1.623 | 38.696 |
| 14 | 1.44 | 3,597,144 | 707,007 | 176,136 | 4.897 | 24.913 |
| 15 | 2.80 | 4,846,883 | 1,864,521 | 472,361 | 9.746 | 25.334 |
| 16 | 3.08 | 4,867,234 | 2,310,199 | 191,622 | 3.937 | 8.295 |
| 17 | 5.52 | 4,254,413 | 2,213,481 | 68,712 | 1.615 | 3.104 |
| 18 | 4.16 | 4,024,488 | 2,178,338 | 5,131 | 0.128 | 0.236 |
| 19 | 6.28 | 5,439,333 | 977,432 | 20,083 | 0.369 | 2.055 |
| 20 | 2.08 | 4,427,061 | 554,782 | 386,973 | 8.741 | 69.752 |
| 21 | 20.92 | 5,532,049 | 2,416,938 | 8,502 | 0.154 | 0.352 |
| 22 | 1.52 | 5,095,586 | 1,580,950 | 48,192 | 0.946 | 3.048 |
| 23 | 4.20 | 3,256,721 | 210,335 | 9,948 | 0.306 | 4.730 |
| 24 | 9.04 | 3,051,553 | 66,996 | 40,791 | 1.337 | 60.886 |
| 25 | 2.84 | 4,585,925 | 3,403 | 1,058 | 0.0231 | 31.090 |
| 26 | 6.68 | 3,979,144 | 72,849 | 4,952 | 0.124 | 6.798 |
| 27 | 6.80 | 5,078,913 | 1,648,599 | 1,188,868 | 23.408 | 72.114 |
| 28 | 2.72 | 3,476,555 | 852,398 | 582,936 | 16.768 | 68.388 |
| 29 | 9.56 | 4,157,644 | 18,531 | 7,832 | 0.188 | 42.264 |
| 30 | 13.92 | 5,022,004 | 781,730 | 341,691 | 6.804 | 43.710 |
| 31 | 3.46 | 2,807,607 | 111,977 | 68,369 | 2.435 | 61.056 |

^a^Concentration of RNA in the samples.

^b^Percentage of reads from *V. cholerae* relative to the total reads.

^c^Percentage of reads from *V. cholerae* relative to the total bacterial reads.

Supplementary Table S5 | Data from the metagenomic sequencing analysis of DNA samples extracted from the stools of 22 diarrheal patients in which the etiological agent of the diarrhea was not detected.

| DNA sample | | | | | | | | | | |  |
| --- | --- | --- | --- | --- | --- | --- | --- | --- | --- | --- | --- |
| Sample No. | Total read | Read obtained after quality filtering^a^ | Total DNA read from bacteria | Ratio of read from bacteria per read after quality filtering (%) | DNA read from *V. cholerae* | DNA read from *ctxA*^b^ | Ratio of read from *V. cholerae* to total DNA read (%)^c^ | Ratio of read from *V. cholerae* to read obtained after quality filtering (%) | Ratio of read from *V. cholerae* to bacterial DNA read (%)^d^ | Ratio of read from *ctxA* to total DNA read (%)^e^ | |
| 1001 | 6,557,095 | 6,553,315 | 993,445 | 15.159 | 210 | 0 | 0.003 | 0.003 | 0.02114 | 0.00000 | |
| 1002 | 7,130,926 | 7,130,125 | 3,195,810 | 44.821 | 193 | 0 | 0.003 | 0.003 | 0.00604 | 0.00000 | |
| 1003 | 6,541,579 | 6,529,423 | 85,689 | 1.312 | 13 | 0 | 0.000 | 0.000 | 0.01517 | 0.00000 | |
| 1004 | 8,199,039 | 8,187,795 | 56,731 | 0.693 | 16,244 | 3 | 0.198 | 0.198 | 28.63338 | 0.00004 | |
| 1005 | 5,724,307 | 5,714,229 | 737,966 | 12.915 | 124 | 0 | 0.002 | 0.002 | 0.01680 | 0.00000 | |
| 1006 | 7,232,105 | 7,229,398 | 313,909 | 4.342 | 734 | 1 | 0.010 | 0.010 | 0.23383 | 0.00001 | |
| 1007 | 5,602,932 | 5,586,668 | 14,544 | 0.260 | 108 | 0 | 0.002 | 0.002 | 0.74257 | 0.00000 | |
| 1008 | 5,524,335 | 5,510,299 | 100,504 | 1.824 | 21 | 0 | 0.000 | 0.000 | 0.02089 | 0.00000 | |
| 1009 | 5,481,552 | 5,481,247 | 223,832 | 4.084 | 35 | 0 | 0.001 | 0.001 | 0.01564 | 0.00000 | |
| 1010 | 7,409,004 | 7,400,569 | 376,807 | 5.092 | 275,326 | 75 | 3.716 | 3.720 | 73.06818 | 0.00101 | |
| 1011 | 6,822,486 | 6,821,453 | 1,604,752 | 23.525 | 98 | 0 | 0.001 | 0.001 | 0.00611 | 0.00000 | |
| 1012 | 8,270,410 | 8,269,495 | 4,080,076 | 49.339 | 342 | 0 | 0.004 | 0.004 | 0.00838 | 0.00000 | |
| 1013 | 7,012,036 | 7,008,376 | 682,816 | 9.743 | 266 | 0 | 0.004 | 0.004 | 0.03896 | 0.00000 | |
| 1014 | 7,143,019 | 7,141,545 | 3,220,304 | 45.093 | 207 | 0 | 0.003 | 0.003 | 0.00643 | 0.00000 | |
| 1015 | 6,306,750 | 6,304,827 | 2,739,692 | 43.454 | 358 | 0 | 0.006 | 0.006 | 0.01307 | 0.00000 | |
| 1016 | 6,996,301 | 6,993,168 | 2,837,415 | 40.574 | 298 | 0 | 0.004 | 0.004 | 0.01050 | 0.00000 | |
| 1017 | 5,033,308 | 5,030,164 | 1,828,615 | 36.353 | 41,722 | 6 | 0.829 | 0.829 | 2.28162 | 0.00012 | |
| 1018 | 6,454,262 | 6,448,853 | 1,211,526 | 18.787 | 33,603 | 5 | 0.521 | 0.521 | 2.77361 | 0.00008 | |
| 1019 | 4,274,302 | 4,273,103 | 164,597 | 3.852 | 5,067 | 0 | 0.119 | 0.119 | 3.07843 | 0.00000 | |
| 1020 | 4,150,430 | 4,150,388 | 127,286 | 3.067 | 10 | 0 | 0.000 | 0.000 | 0.00786 | 0.00000 | |
| 1021 | 4,055,990 | 4,045,493 | 16,373 | 0.405 | 14 | 0 | 0.000 | 0.000 | 0.08551 | 0.00000 | |
| 1022 | 3,138,738 | 3,138,390 | 1491948 | 47.539 | 1282 | 0 | 0.041 | 0.041 | 0.08593 | 0.00000 | |

^a^Number of the read after quality filtering was obtained by the trimming.

^b^*ctxA*; the gene for A subunit of cholera toxin.

^c^Percentage of reads from *V. cholerae* relative to the total reads.

^d^Percentage of reads from *V. cholerae* relative to the total bacterial reads.

^e^Percentage of reads from *ctxA* relative to the total reads.

Supplementary Table S6 | Data from the metagenomic sequencing analysis of RNA samples extracted from the stools of 22 diarrheal patients in which the etiological agent of the diarrhea was not detected.

| RNA sample | | | | | |
| --- | --- | --- | --- | --- | --- |
| Sample number | Total reads | Total RNA reads from bacteria | RNA reads from *V. cholerae* | Ratio to the total RNA (%)^a^ | Ratio to the total bacterial RNA　(%)^b^ |
| 1001 | 3,419,556 | 247,374 | 72 | 0.002 | 0.0291 |
| 1002 | 5,152,296 | 1,802,905 | 1,004 | 0.020 | 0.0557 |
| 1003 | 6,278,923 | 1,040,652 | 158 | 0.003 | 0.0152 |
| 1004 | 3,916,149 | 143,222 | 76,602 | 1.9561 | 53.4848 |
| 1005 | 4,982,547 | 2,025,904 | 717 | 0.014 | 0.0354 |
| 1006 | 5,737,430 | 73,873 | 16 | 0.000 | 0.0217 |
| 1007 | 5,272,539 | 101,054 | 88 | 0.002 | 0.0871 |
| 1008 | 5,130,880 | 167,630 | 50 | 0.001 | 0.0298 |
| 1009 | 5,371,329 | 2,656,148 | 942 | 0.018 | 0.0355 |
| 1010 | 5,481,782 | 1,924,142 | 977,681 | 17.835 | 50.8113 |
| 1011 | 5,575,071 | 2,095,519 | 575 | 0.010 | 0.0274 |
| 1012 | 2,635,059 | 178,963 | 709 | 0.0270 | 0.3962 |
| 1013 | 5,289,713 | 753,662 | 156 | 0.003 | 0.0207 |
| 1014 | 5,736,397 | 2,072,345 | 129 | 0.002 | 0.0062 |
| 1015 | 5,432,714 | 707,201 | 367 | 0.007 | 0.0519 |
| 1016 | 5,038,127 | 190,592 | 107 | 0.002 | 0.0561 |
| 1017 | 3,922,936 | 1,327,838 | 63,847 | 1.628 | 4.8083 |
| 1018 | 4,463,794 | 980,966 | 149,885 | 3.358 | 15.2793 |
| 1019 | 4,216,181 | 521,269 | 91 | 0.002 | 0.0175 |
| 1020 | 5,256,849 | 1,228,302 | 369 | 0.007 | 0.030 |
| 1021 | 5,708,662 | 442,552 | 37 | 0.001 | 0.0084 |
| 1022 | 5,095,524 | 1,106,998 | 23,472 | 0.461 | 2.1203 |

^a^Percentage of reads from *V. cholerae* relative to the total reads.

^b^Percentage of reads from *V. cholerae* relative to the total bacterial reads.
